# Effects of intravaginal progesterone devices and Booroola fecundity (*FecB*) gene on the ovarian responses and reproductive indices of Qezel ewes following estrus synchronization and laparoscopic insemination during the breeding season

**DOI:** 10.5194/aab-68-425-2025

**Published:** 2025-07-01

**Authors:** Mohammad Allah-Dadi, Mohsen Eslami, Farhad Farrokhi-Ardabili, Sina Bahmani

**Affiliations:** 1 Department of Theriogenology, Faculty of Veterinary Medicine, Urmia University, Urmia, Iran; 2 Department of Animal Sciences, Faculty of Agriculture, Urmia University, Urmia, Iran

## Abstract

The ovarian responses concerning types of vaginal devices in fat-tailed ewes carrying the Booroola fecundity gene (*FecB*) has not been studied in detail. Evaluation of the ovulatory responses and distributions following estrus synchronization with controlled internal drug release (CIDR) or a vaginal sponge in ewes carrying *FecB* compared to non-carriers and reproductive indices after laparoscopic insemination (LI) are the aims of the current study. Estrus was synchronized using CIDR ( n=16 ) or medroxyprogesterone acetate (MPA) sponge ( n=15 ) insertion (day - 14) and an eCG injection on device removal (day 0). Laparoscopic insemination was performed, and the ovarian structures were measured intensively using a transvaginal probe after - 14 and 0 d as well as after 36–38 and 48–51 h and continued every 4 h until 75 h after the eCG treatment. The progesterone amounts were measured in sera samples. The initiation and termination of ovulations happened earlier in CIDR-receiving ewes and *FecB* carriers compared to MPA-sponge-treated ewes and non-carriers, respectively ( P<0.05 ). Progesterone concentrations were higher in the CIDR group compared to MPA sponge group on day 0. Device types did not affect the sizes of ovulatory follicles, corpora lutea diameters, and reproductive indices ( P>0.05 ), while the twining rate, litter size, and reproductive rate were greater in *FecB* carriers compared to non-carriers ( P<0.05 ). Ovulation synchronization with MPA sponge with an eCG delayed the ovulation but did not influence the conception rate compared to CIDR-treated ewes. In spite of the smaller sizes of the ovulatory follicles and subsequent corpora lutea, higher reproductive outcomes were acquired in the *FecB* carriers compared to non-carrier fat-tailed ewes following LA.

## 1 Introduction

Estrus synchronization protocols directly affect the income of the livestock breeders by improving reproductive performance (Abecia et al., 2011; Qasemi-Panahi et al., 2016). Many estrus synchronization protocols have been developed for sheep, and one of the most successful is based on the temporary suppression of the estrus with the help of progestins (Robinson, 1965; Maxwell and Barnes, 1986; Gourley and Riese, 1990; Bretzlaff and Romano, 2001; Gordon, 2017). Progestins have an inhibitory effect on luteinizing hormone (LH) release from the anterior pituitary, which prevents the growth and final maturation of ovarian antral follicles and ovulation. After progestin cessation, the onset of estrus and ovulation occurs at a predictable time (Dogan et al., 2004). Controlled internal drug release (CIDR) and medroxyprogesterone acetate (MPA) or flugestone acetate (FGA) intravaginal sponges have been successfully used for synchronization of estrus and, consequently, natural breeding or laparoscopic artificial insemination (LAI) in ewes (Luther et al., 2007). It has been shown that an injection of equine chorionic gonadotropin (eCG) after progesterone cessation or removal increases the ovulation rate, improves fertility, and also reduces the variations in the initiation of estrus and ovulation (Rensis and López-Gatius, 2014; Qasemi-Panahi et al., 2016).

Artificial insemination (AI) is an effective tool to overcome the limitation of seasonal reproduction in small ruminants (Abecia et al., 2012). The development of AI makes it possible to use the semen of the superior rams to impregnate ewes and maximize genetic development (Salamon and Maxwell, 2000). A prerequisite for successful AI in sheep is accurate estrus detection (several times a day), which is an impractical task. In addition, the fertility rate depends on the synchrony between the ovulation and insemination time (Reyna et al., 2007). In order to overcome the mentioned problem, fixed-time artificial insemination (FTAI) is performed following the synchronization of estrus and ovulation with the use of hormonal treatments. However, variability in ovulatory responses, even with an advanced understanding of ovarian follicular development and performing estrus synchronization, persists as a major limiting factor in increasing the conception rates, especially after AI (Gordon, 1983; Walker et al., 1989).

The Booroola fecundity (*FecB*) gene, also known as bone morphogenetic protein receptor type 1B (BMPR1B), was identified in the Booroola Merino sheep as the major gene responsible for prolificacy (Mulsant et al., 2001; Souza et al., 2001; Wilson et al., 2001). It was verified that the *FecB* gene increases the ovulation rate and, consequently, the litter size in ewes (Davis, 2004). Researchers have tried to screen the BMPR1B mutation and its responsibility for higher prolificacy in other prolific sheep breeds (Liu et al., 2003; Davis, 2005; Yao et al., 2006; Kumar et al., 2008; Hua and Yang, 2009; Mahdavi et al., 2014; Qi et al., 2020). Moreover, researchers have tried to introgress the *FecB* in some less prolific breeds to improve the prolificacy rates (Mishra et al., 2007; Chu et al., 2007; Hua and Yang, 2009). Reviewing the literature revealed that the determination of ovulation time following estrus synchronization using different intravaginal progesterone devices in B allele carrier (B + ) and non-carrier ewes has not been published about. Therefore, the current study aimed to evaluate the effects of the introgression of the *FecB* allele (as B + ) on the distribution of ovulations (every 3–4 h with transvaginal ultrasonography) following estrus synchronization with two types of vaginal containing progestogens devices (CIDR or MPA sponge) and reproductive outcomes after fixed-time laparoscopic insemination (FTLI) in Qezel ewes during the breeding season.

## 2 Materials and methods

### 2.1 Experimental animals and location

In total, 31 fat-tailed Qezel ewes (28.37 ± 2.02 months of age; 61.83 ± 3.10 kg body weight) were included in the study during the breeding season (October–November). A total of 13 ewes carried the *FecB* gene as heterozygous, and the remainder (18) did not have the *FecB* gene. The ewes were kept under uniform nutritional and management condition at the small ruminant farm belonging to the Agriculture Faculty of Urmia University, West Azerbaijan, Iran. Before starting the experiment, scheduled vaccination was done.

### 2.2 Estrus synchronization

Estrus was synchronized by CIDR (Eazi-Breed CIDR, Zoetis, Belgium; carrying 0.3 g endogenic progesterone) or MPA vaginal sponge (Sponjavet, Hipra, Spain; 60 mg) insertion (day - 14) for 14 d, accompanied with eCG (Gonaser, Hipra, Spain; 400 IU) injection on device removal (day 0 of the experiment). A total of 16 (6 ewes carrying *FecB* and 10 ewes without *FecB*) and 15 (7 ewes carrying *FecB* and 8 ewes without *FecB*) ewes were allocated in CIDR and vaginal sponge groups, respectively. Intensive ovarian ultrasonography using the transvaginal approach was performed until the ovulation was confirmed. FTLI with freshly diluted semen samples was done 57 ± 0.5 and 64 ± 0.5 h after eCG injection in CIDR- and MPA-inserted ewes, respectively.

### 2.3 Ovarian examination using a transvaginal probe

The ovarian structures were scanned by a real-time, B-mode ultrasonography equipped with a 9 MHz transvaginal transducer (Emperor, EMP 830Vet, China) at - 28 (to detect the presence of corpus luteum), - 21 (to confirm the presence of corpus luteum), and - 14 d (device insertion). A total of 54 ewes were examined, and if the corpus luteum was present on the ovaries - 28 and - 21 d of the experiment, the ewes were considered cyclic and entered into the experiment. After device withdrawal, an intensive ovarian examination was performed 36–38 and 48–51 h and continued every 4 h until 75 h after eCG injection to confirm the time of ovulation and record the ovarian follicle sizes and the number of ovulated follicles. Vaginal ultrasonographic examination was performed in the ewes in standing position. Details of ovarian follicles (at day 0 onwards) such as their numbers and diameters (greater than 1 mm) were recorded for every ewe and tabulated for growth. The ovulation was detected and confirmed (two consecutive examinations) by the disappearance of a large, growing follicle that was tracked several times followed by the formation of the corpus luteum in the referred ovary. Furthermore, the ovarian examination was done on day + 11 (9 d after insemination) to record the number and diameters of the corpora lutea.

### 2.4 Semen samples and laparoscopic insemination

An artificial vagina (IMV, France) was used to collect the semen samples from the fertile ram (carrying *FecB* as homozygous) in the presence of an estrus ewe. The mean concentration and total and forward progressive motility of the two collected samples were 3.17 × 109 spermatozoa mL, 98.56 %, and 92.18 %, respectively. The motility was assessed by Computer-Assisted Sperm Analysis (CASA) (video test; Test Sperm 3.2, Saint Petersburg, Russia) equipped with a warm stage and a phase-contrast microscope (Labomed, Labomed Inc., Culver City, CA, USA) after dilution with a prepared extender (15 µ L sample + 985 µ L extender). After the initial assessment, the semen samples were diluted at a rate of 300 × 10^6^ spermatozoa mL^−1^ with tris-citric acid-fructose and plasma egg yolk, according to the previous described protocol (Mortazavi et al., 2020). Ewes were inseminated through a laparoscopic approach 56 ± 0.5 and 64 ± 0.5 h after CIDR and MPA sponge removal with an eCG injection, respectively. Each uterine horn was inseminated with 0.25 mL extended semen. The light source and trocars (5 mm) were purchased from the Storz company (Germany). The semen sampling process, transvaginal examination, and laparoscopic insemination were verified by the care committee of Urmia University (IR-UU-AEC-3/28).

### 2.5 Progesterone concentrations

In order to measure the progesterone (P4) concentrations, the jugular vein was punctured using a needle, and the vacuum tube (VACUETTE, Bio-one GmbH, Austria) was attached to collect the blood sample. Blood samples of ewes were collected on days - 14, 0, + 2, and + 11 during the experiment. Samples were centrifuged at 4000 g for 15 min to separate the sera parts. Sera samples were preserved at - 20 °C until P4 assay. The P4 concentrations were measured using a specific ELISA kit (sheep progesterone ELISA kit, ZellBio, Germany) and ELISA reader at 450 nm.

### 2.6 Reproductive outcomes

The intravaginal ultrasonographic examination was performed to detect the presence and number of alive embryos 38 d after insemination. The percent of conception rate (number of pregnant ewes on day 38 / number of inseminated ewes), twining or tripling rate (number of pregnant ewes having two or three viable embryos / total number of pregnant ewes), and reproductive rate (number of lambs born / total number of inseminated ewes) were calculated concurrently with the litter size (number of total lambs / number of ewes lambing) analyzed between groups.

### 2.7 Genotyping

The genotype at the *FecB* locus was validated by the polymerase chain reaction–restriction fragment length polymorphism (PCR-RFLP) method (Wilson et al., 2001).

### 2.8 Statistical analysis

The effect of vaginal devices and *FecB* on the sizes of ovulated follicles, ovulation time, number of corpora lutea, and P4 concentrations were analyzed using two-way ANOVA. The conception and twining rates were assessed using the chi-square test. The litter size and reproductive rate were analyzed between groups using the Mann–Whitney rank sum test. The SigmaStat software (Version 3.5; Chicago, IL) was used to analyze the data statistically. A p value lower than 0.05 was considered significant, and the p value between 0.05 and 0.10 was considered to tend to be significant.

## 3 Results

One ewe was excluded from the vaginal sponge group due to respiratory disease and fever on day + 2. The ovulation was recorded in all remaining ewes ( n=30 ). The initiation (51–55 h vs. 63–67 h; P<0.001 ) and termination (63–67 h vs. 67–71 h; P<0.001 ) of ovulations were different in CIDR-treated compared to sponge-receiving ewes after devices removal and eCG administration (Table 1).

**Table 1:** The number of ewes ovulating for the first time and total number of ovulated follicles within different time spans following ovulation synchronization with CIDR ( n=16 ewes) or MPA vaginal sponges ( n=14 ewes) in combination with an eCG injection (400 IU) in *FecB* carrier ( n=12 ) and non-carrier ( n=18 ) Qezel ewes.

		Time spans after device removal + eCG injection							
		36–38	48– <51	51– <55	55– <59	59– <63	63– <67	67– <71	71–75
First ovulation	CIDR ( n=16 )	0	0	1	7	5	3	0	0
	Sponge ( n=14 )	0	0	0	0	0	8	6	0
Total number of	CIDR (30)	0	0	3	14	8	5	0	0
ovulated follicles	Sponge (27)	0	0	0	0	0	15	12	0
Total ovulated follicles	Carrying *FecB* (17)	0	0	3	11	3	0	0	0
in CIDR group	NC (13)	0	0	0	3	5	5	0	0
Total ovulated follicles	Carrying *FecB* (15)	0	0	0	0	0	8	7	0
in sponge group	Non-carriers (12)	0	0	0	0	0	7	5	0

CIDR: controlled internal drug releasing devices, MPA: medroxyprogesterone acetate, eCG: equine chorionic gonadotropin, IU: international unit, NC: non-carriers. A total of 6 and 6 ewes carrying *FecB* and 10 and 8 non-carrier ewes were allocated to CIDR and sponge groups, respectively.

A total of 17 and 15 follicles and 13 and 12 follicles were ovulated from the ewes carrying *FecB* and non-carriers, in the CIDR- and sponge-treated ewes, respectively. The vaginal device types and presence of *FecB* were effective at the time of first and all ovulations (Table 2). CIDR-treated ewes and *FecB* carriers ovulated earlier (first and all ovulations) compared to sponge-receiving ewes and non-carriers, respectively ( P<0.05 ; Table 2).

**Table 2:** Ovulatory performances of fat-tailed Qezel ewes following ovulation synchronization with CIDR ( n=16 ewes) or MPA vaginal sponges ( n=14 ewes) in combination with an eCG injection (400 IU) in *FecB* carriers ( n=12 ) and non-carriers ( n=18 ) during the breeding season.

	CIDR				MPA sponge					
Indices	Total	*FecB*	NC	P value	Total	*FecB*	NC	P value	P value	P value
		carriers		(*FecB* vs. NC)		carriers		(*FecB* vs. NC)	(CIDR vs. sponge)	(*FecB* vs. NC)
Number ofovulatory follicles	30	17	13	0.001	27	15	12	0.02	0.91	<0.001
Mean time of firstovulation (h)	60.04	57.66	61.40	<0.001	66.71	67.02	66.50	0.512	0.001	0.044
Mean time of allovulation (h)	59.40	57.64	61.84	<0.001	66.77	67.86	66.66	0.629	0.001	0.008
Mean size of ovulatory follicles (mm)	5.95	5.27	7.20	<0.001	6.01	5.46	6.70	0.007	0.525	<0.001
Number of corpusluteum	30	17	13	0.001	27	15	12	0.02	0.91	<0.001
Mean size of corpusluteum (mm)	11.22	10.38	12.32	0.003	10.56	9.75	11.57	0.006	0.11	<0.001
Mean interval from first to last ovulations(min–max)	0	0	0	–	0.57 (0.4)	0	1 (0–4)	–	–	–

CIDR: controlled internal drug releasing devices, MPA: medroxyprogesterone acetate, eCG: equine chorionic gonadotropin, IU: international unit, NC: non-carriers. A total of 6 and 6 ewes carrying *FecB* and 10 and 8 non-carrier ewes were allocated to the CIDR and sponge groups, respectively.

Device types did not affect the mean sizes of ovulatory follicles, while *FecB* carriers ovulated at smaller sizes compared to non-carrier ewes (5.31 vs. 6.95 mm; Table 3). Furthermore, the mean sizes of corpora lutea were smaller in *FecB* carriers compared to non-carriers (10.02 vs. 11.95 mm; Table 3). Moreover, mean sizes of ovulatory follicles ( P<0.001 ) and corpora lutea ( P=0.016 ) were smaller in multi-ovulatory ewes compared to single-ovulatory ewes (Table 4).

**Table 3:** Ovulatory performances, progesterone (P4) concentrations, and reproductive performances of fat-tailed Qezel ewes carrying *FecB* ( n=12 ) compared to non-carriers ( n=18 ) following ovulation synchronization with CIDR or MPA vaginal sponges in combination with an eCG injection (400 IU) during the breeding season.

	*FecB* carriers ( n=12 )	Non-carriers ( n=18 )	P value (*FecB* vs. NC)
Indices			
Number of ovulatory follicles	32	25	<0.001
Mean sizes of ovulatory follicles (mm)	5.31	6.95	<0.001
Mean sizes of corpus luteum (mm)	10.02	11.95	<0.001
Mean P4 (ng mL^−1^) concentrations on day - 14	5.67 ± 1.41	2.34 ± 0.48	<0.001
Mean P4 (ng mL^−1^) concentrations on day + 11	8.82 ± 0.57	5.17 ± 0.48	<0.001
Conception rate % ( n/n )	91.66 ( 11/12 )	61.11 ( 11/18 )	0.099
Twinning or tripling rate % (n/n)	100 ( 11/11 )	27.27 ( 3/11 )	0.09
Litter size	2.36	1.27	0.001
Reproductive rate	2.16	0.077	0.001

CIDR: controlled internal drug releasing devices, MPA: medroxyprogesterone acetate, eCG: equine chorionic gonadotropin, IU: international unit, NC: non-carriers, P4: progesterone. Laparoscopic insemination was performed 56 ± 0.5 and 64 ± 0.5 h after CIDR and sponge removal with an eCG injection, respectively.

**Table 4:** Ovulatory performances of single-ovulatory (SO) and multi-ovulatory (MO) fat-tailed Qezel ewes following synchronization of ovulation with CIDR ( n=16 ewes) or MPA vaginal sponges ( n=14 ewes) in combination with an eCG injection (400 IU) during the breeding season.

	CIDR				MPA sponge					
Indices	Total	MO	SO	P	Total	MO	SO	P	C vs. S	MO vs. SO
	( n=16 )	( n=9 )	( n=7 )	(MO vs. SO)	( n=14 )	( n=9 )	( n=5 )	(MO vs. SO)		
Number of ovulatoryfollicles	30	23	7	<0.001	27	22	5	<0.001	0.91	<0.001
Mean time of ovulation	59.40	59.13	60.57	0.256	66.77	66.46	78.20	0.276	0.001	0.119
Mean size of the ovulatory follicles (mm)	5.95	5.39	7.81	<0.001	6.01	5.96	6.2	0.686	0.193	0.001
Mean size of the corpus luteum (mm)	11.22	10.85	12.44	0.049	10.56	10.30	11.71	0.133	0.287	0.016

CIDR: controlled internal drug releasing devices, MPA: medroxyprogesterone acetate, eCG: equine chorionic gonadotropin, IU: international unit, NC: non-carriers.

**Figure 1:**
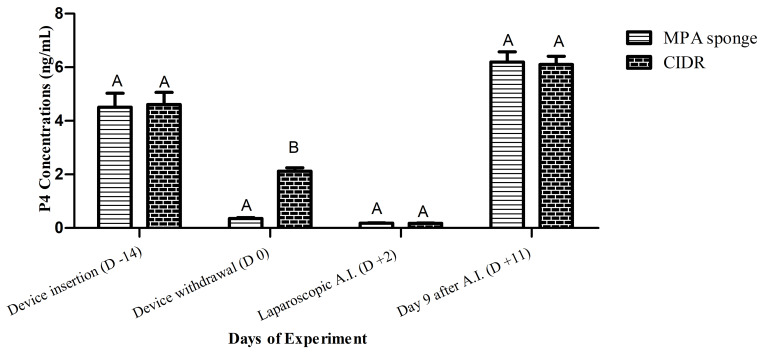
Mean progesterone (P4; ng mL^−1^) concentrations of CIDR and sponge-treated ewes on device insertion ( - 14), device withdrawal (0), laparoscopic AI ( + 2), and day 9 after insemination (day + 11 of the experiment). Higher P4 amounts were detected in CIDR-receiving group at the time of device removal compared to sponge-primed females.

Mean P4 concentrations did not differ between CIDR and MPA-sponge-treated females on days - 14, + 2, and + 11 (Fig. 1). Higher P4 amounts were recorded in CIDR-treated ewes compared to MPA-sponge-treated females on day 0 ( P<0.001 ; Fig. 1). Furthermore, greater amounts of P4 were recorded ( P<0.001 ) in *FecB* carriers compared to non-carrier females on days - 14 (5.67 ± 1.41 vs. 2.34 ± 0.48 ng mL^−1^) and + 11 (8.82 ± 0.57 vs. 5.17 ± 0.48 ng mL^−1^; Table 3).

The conception rate was 75 % and 71.42 % in CIDR and sponge-receiving groups following insemination after 56 ± 0.5 and 64 ± 0.5 h, respectively (Table 5; P=0.84 ). *FecB* carriers ( 11/12=91.66 %) tend to have a higher conception rate compared to non-carriers ( 11/18=61.11 %; P=0.099 ; Table 3). Twinning or tripling rate, litter size, and reproductive rate did not differ between CIDR and sponge-receiving groups, while *FecB* carrier ewes showed greater twinning or tripling rates (100 vs. 27.27 %), litter size (2.36 vs. 1.27), and reproductive rate (2.16 vs. 0.77) compared to non-carrier females (Table 3; P<0.05 ).

**Table 5:** Reproductive performances of *FecB* carrier ( n=12 ) and non-carrier ( n=18 ) fat-tailed Qezel ewes following ovulation synchronization with CIDR ( n=16 ) or MPA vaginal sponges ( n=14 ) in combination with an eCG injection (400 IU) during the breeding season.

	CIDR			Sponge			P value	P value
Indices	Total	*FecB*	NC	Total	*FecB*	NC	(CIDR vs. sponge)	(*FecB* vs. NC)
Conception rate (%)	75	100	60	71.42	83.33	62.50	0.84	0.099
Twinning or triplingrate (%)	66.66	100	33.33	60	100	20	0.90	0.09
Litter size	1.75	2.16	1.33	1.90	2.60	1.20	0.748	0.001
Reproductive rate	1.31	2.16	0.8	1.35	2.16	0.75	0.983	0.001

CIDR: controlled internal drug releasing devices, MPA: medroxyprogesterone acetate, eCG: equine chorionic gonadotropin, IU: international unit, NC: non-carriers. Laparoscopic insemination was performed 56 ± 0.5 and 64 ± 0.5 h after CIDR and sponge removal with an eCG injection, respectively.

## 4 Discussion

The present study was designed to examine the effects of the introgression of *FecB* into genome of a less prolific breed (Qezel) on the timing and distribution of ovulations (assessed by serial intensive transvaginal ultrasonographic scanning) following estrus synchronization with vaginal loaded progestogen devices (CIDR vs. MPA sponge) with eCG and reproductive outcomes after FTLI. Results of the current study indicated that (1) CIDR-treated ewes (*FecB* carriers and non-carriers) ovulated earlier compared to MPA-receiving ewes after device removal with an eCG injection; (2) in the CIDR group, *FecB* carriers initiated and terminated the ovulation earlier compared to non-carriers after device removal with an eCG injection; (3) sizes of ovulated follicles and subsequent corpora lutea were smaller in *FecB* carriers, while the number of ovulatory follicles and progesterone concentrations (in the luteal phase) were more remarkable compared to non-carriers; and (4) in total, the reproductive performance was significantly improved by the introgression of *FecB* into the genome of Qezel ewes.

The *FecB* gene has been identified in the Booroola Merino and some other prolific sheep breeds (Davis et al., 2006; Guan et al., 2007; Qi et al., 2020). Under an intensive management system, improvement in prolificacy is a desirable trait for dairy and non-dairy sheep breeds. Then, crossbreeding was programmed and performed in less to moderate prolific breeds all over the world, such as Assaf, Awassi, Garole, Small Tail Han, Huyang, Cele, and Duolang (Mishra et al., 2007; Chu et al., 2007; Kumar et al., 2008; Hua and Yang, 2009). This crossbreeding increased the prolificacy in the generations that follow (Guan et al., 2007; Chu et al., 2007; Gootwine et al., 1995, 2003, 2008). However, a previous study indicated that the prolificacy of B + ewes (heterozygous situation) was higher than BB (homozygous situation) ewes (Farquhar et al., 2006), so the BB ewes were not included in the current study. Studies revealed that *FecB* introgressed ewes had greater numbers of ovulated follicles, while their sizes were smaller compared to ++ (non-carrier) ewes (Souza et al., 1994, 1997). However, the mentioned authors evaluated the ovarian follicles (number and sizes) by laparoscopic examination or transabdominal ultrasonographic scanning every 12 h intervals (Driancourt et al., 1985; Souza et al., 1994, 1997). The efficacy of laparoscopic lenses in the evaluation of the exact time and sizes of ovulations was under question. Estrus synchronization with the utilization of vaginal devices with eCG has usually resulted in an acceptable pregnancy rate following natural breeding. However, the variation in ovulation time is the main restricting factor in the formation of zygotes and establishing the pregnancy following FTAI. The external and internal factors affect the ovulation time and its variances in sheep herds (Gibbons et al., 1999; Viñoles et al., 1999; Salehi et al., 2010). Even by minimizing these factors, the ovulation time is varied among ewes (Salehi et al., 2010). Limited studies were reported about the ovulation distributions after progesterone device withdrawal. The authors hypothesized that the ovulation distributions would be different following synchronization with MPA vaginal sponges with eCG compared to CIDR with eCG in *FecB* carriers and non-carrier Qezel ewes and consequently affect the reproductive outcomes after FTLI. Using intensive serial transvaginal ultrasonographic examination, we confirmed the greater numbers of ovulated follicles at smaller sizes in *FecB* carriers compared to non-carriers (5.31 vs. 6.95 mm). Furthermore, the mean time of first and all ovulations (after device removal and eCG injection) was lower in *FecB* carriers compared to non-carriers. This is an important finding, especially for programming the time of insemination with frozen/thawed or even fresh semen samples. Furthermore, results of the present study indicated that CIDR-treated ewes completed the ovulation earlier (concerning eCG injection) compared to MPA-sponge treated ewes (59.74 vs. 66.91 h), which is in accordance with the previous reports (Walker et al., 1989; Husein and Kridli, 2002; Bartlewski et al., 2015). Differences in the molecular characteristics, the physical form, and consequently varying absorbances during and after withdrawal, half-life, and clearance rate between MPA and CIDR (endogenous progesterone) devices are the proposed reasons for the mentioned findings (Ryan and Rosner, 2001; Husein and Kridli, 2002; Menchaca et al., 2007; Bartlewski et al., 2015).

In the present experiment, progesterone concentrations on device withdrawal were significantly higher in CIDR-treated (2.12 ± 0.16 ng mL^−1^) compared to MPA-sponge-treated ewes (0.35 ± 0.08 ng mL^−1^). It seems that a progesterone assay kit used in the present study could not cross-react with the exogenous progestogens released from the MPA vaginal sponge. This finding is compatible with the previous report (Husein and Kridli, 2002). Furthermore, higher amounts of progesterone in *FecB* carriers compared to non-carrier ewes on days - 14 and + 11 seems to be the case due to greater numbers of corpora lutea in the B + ewes.

Fixed-time insemination has numerous advantages in the management of ewe breeding; however, one of the main associated problems is the accurate estimation of ovulation time, which is necessary for performing insemination in a timely manner and hence maximizing fertility (Romano, 1996). Controversial reports have been released about the correlation between ovulation distributions, time of AI, and conception rate. Higher conception rate (63 % vs. 38 %) was reported following insemination 44 h compared to 68 h after FGA removal (Maxwell et al., 1993), while a lower conception rate was reported after 48 h compared to insemination after 60 h (55 % vs. 46 %; Maxwell, 1986). Furthermore, insemination 48 h after CIDR withdrawal resulted in a higher conception rate compared to MPA-sponge-treated ewes (73 % vs. 52 %; Fukui et al., 1993). The proposed time span for laparoscopic insemination following synchronization is 48–72 h after MPA sponge withdrawal with eCG administration. Insemination before (24–36 h) or after (78 h) the recommended period resulted in a lower conception rate (Maxwell et al., 1984; Maxwell, 1986; Jabbour and Evans, 1991). Due to differences in the initiation of estrus and ovulation following priming with endogenous (CIDR) and exogenous (MPA sponge) progestogen devices, in the present experiment, ewes were inseminated 57 ± 0.5 and 64 ± 0.5 h after eCG injection in CIDR- and MPA-sponge-treated, respectively. Results of the present experiment indicated that the conception rate did not differ between CIDR- and sponge-receiving ewes (75 vs. 71.42 %), and a non-significant increase was observed in *FecB* carriers (91.66 %, 11/12 ) compared to non-carrier ewes (61.11 %, 11/18 ; P=0.099 ). However, the twinning rate, litter size, and reproductive rate were significantly higher in *FecB* carriers compared to non-carrier ewes, which is consistent with other research (Meyer et al., 1994; Chu et al., 2007; Guan et al., 2007; Mahdavi et al., 2014; Qi et al., 2020). A recent study indicated that the conception rate among BB, B + , and ++ genotypes was similar 60 d after AI in sheep; however, the litter size was affected by B allele (Qi et al., 2020). Controversial findings were reported about the role of *FecB* in the follicle stimulation hormone (FSH) concentrations and finally the number of ovulated follicles (Souza et al., 1997; Hudson et al., 1999; Decuypere et al., 2004; Qi et al., 2020). It was proposed that the *FecB* allele increased the ovulation rate via alterations in the follicular development and oocyte ultrastructure at an early stage (Reader et al., 2012).

## 5 Conclusion

In conclusion, introgression of the *FecB* into the genome of Qezel ewes, previously known as a less prolific fat-tailed breed, increased the number of ovulatory follicles at smaller sizes and consequently improved the reproductive outcomes following synchronization of either CIDR or MPA sponge with eCG. The lower mean time of ovulation (more than 4 h) in *FecB* carriers compared to non-carries in CIDR-treated ewes would be an effective and important factor during FTLI programs. However, synchronization with the MPA sponge overcomes the effects of *FecB* on the mean time of ovulation during the breeding season. Furthermore, it is recommended to conduct insemination earlier (about 6–8 h) of CIDR-receiving compared to MPA-receiving ewes. Determination of the *FecB* genotype in mixed herds would help to adapt the synchronization and insemination protocol.

## Data Availability

The data used and analyzed during this study are available from the corresponding author upon reasonable request.

## References

[bib1.bib1] Abecia JA, Forcada F, González-Bulnes A (2011). Pharmaceutical control of reproduction in sheep and goats. Vet Clin Food Anim Pract.

[bib1.bib2] Abecia JA, Forcada F, González-Bulnes A (2012). Hormonal control of reproduction in small ruminants. Anim Reprod Sci.

[bib1.bib3] Bartlewski PM, Seaton P, Szpila P, Oliveira ME, Murawski M, Schwarz T, Kridli RT, Zieba DA (2015). Comparison of the effects of pretreatment with Veramix sponge (medroxyprogesterone acetate) or CIDR (natural progesterone) in combination with an injection of estradiol-17
β
 on ovarian activity, endocrine profiles, and embryo yields in cyclic ewes superovulated in the multiple-dose Folltropin-V (porcine FSH) regimen. Theriogenology.

[bib1.bib4] Bretzlaff KN, Romano JE (2001). Advance reproductive techniques in goats. Vet Clin North Am Food Anim Pract.

[bib1.bib5] Chu MX, Liu ZH, Jiao CL, He YQ, Fang L, Ye SC, Chen GH, Wang JY (2007). Mutations in BMPR-IB and BMP-15 genes are associated with litter size in Small Tailed Han sheep (Ovis aries). J Anim Sci.

[bib1.bib6] Davis GH (2004). Fecundity genes in sheep, Anim. Reprod. Sci., 82–83, 247–53.

[bib1.bib7] Davis GH (2005). Major genes affecting ovulation rate in sheep. Genet Sel Evol.

[bib1.bib8] Davis GH, Balakrishnan L, Ross IK, Wilson T, Galloway SM, Lumsden BM, Hanrahan JP, Mullen M, Mao XZ, Wang GL, Zhao ZS, Zeng YQ, Robinson JJ, Mavrogenis AP, Papachristoforou C, Peter C, Baumung R, Cardyn P, Boujenane I, Cockett NE, Eythorsdottir E, Arranz JJ, Notter DR (2006). Investigation of the Booroola (FecB) and Inverdale (FecX(I)) mutations in 21 prolific breeds and strains of sheep sampled in 13 countries. Anim Reprod Sci.

[bib1.bib9] Decuypere E, Onagbesan OM, Michels H, Beerlandt G, Peeters R, Bister JL, Pauay R (2004). Gene-specific pituitary gland responsiveness of ovariectomized FecB or FecC carrier and non-carrier ewe crosses with German Mutton Merino, Texel and Suffolk breeds to LHRH before and after oestradiol or progesterone treatments. Small Rumin Res.

[bib1.bib10] Dogan I, Nur Z, Gunay U, Soylu MK, Sonmez C (2004). Comparison of fluorgestone and medroxyprogesterone intravaginal sponges for oestrus synchronization in Saanen does during the transition period. South Africa J Anim Sci.

[bib1.bib11] Driancourt MA, Cahill LP, Bindon BM (1985). Ovarian follicular populations and preovulatory enlargement in Booroola and control Merino ewes. J Reprod Fertil.

[bib1.bib12] Farquhar PA, Dodds KG, Davis GH (2006). Introgression of the Booroola mutation (FecB) leads to hyper-prolificacy in a Romney sheep flock. 8th World Congress on Genetics Applied to Livestock Production.

[bib1.bib13] Fukui Y, Hirai H, Honda K, Hayashi K (1993). Lambing rates by fixed-time intrauterine insemination with frozen semen in seasonally anestrous ewes treated with a progestogen impregnated sponge or CIDR device. J Reprod Dev.

[bib1.bib14] Gibbons JR, Kot K, Thomas DL, Wiltbank MC, Ginther OJ (1999). Follicular and FSH dynamics in ewes with a history of high and low ovulation rates. Theriogenology.

[bib1.bib15] Gootwine E, Bor A, Braw-Tal R, Zenou A (1995). Reproductive performance and milk production of the improved Awassi breed as compared to its crosses with the Booroola Merino. Animal Sci.

[bib1.bib16] Gootwine E, Rozov A, Bor A, Reicher S (2003). Effects of the FecB (Booroola) gene on reproductive and productive traits in the Assaf breed. Proceedings of the International Workshop on Major Genes and QTL in Sheep and Goats.

[bib1.bib17] Gootwine E, Reicher S, Rozov A (2008). Prolificacy and lamb survival at birth in Awassi and Assaf sheep carrying the FecB (Booroola) mutation. Anim Reprod Sci.

[bib1.bib18] Gordon I (1983). Controlled Breeding in Farm Animals.

[bib1.bib19] Gordon I (2017). Reproductive Technologies in Farm Animals.

[bib1.bib20] Gourley DD, Riese RL (1990). Laparoscopic artificial insemination in sheep. Vet Clin North Am Food Anim Pract.

[bib1.bib21] Guan F, Liu SR, Shi GQ, Yang LG (2007). Polymorphism of FecB gene in nine sheep breeds or strains and its effects on litter size, lamb growth and development. Anim Reprod Sci.

[bib1.bib22] Hua GH, Yang LG (2009). A review of research progress of FecB gene in Chinese breeds of sheep. Anim Reprod Sci.

[bib1.bib23] Hudson NL, O'Connell AR, Shaw L, Clarke IJ, McNatty KP (1999). Effect of exogenous FSH on ovulation rate in homozygous carriers or noncarriers of the Booroola FecB gene after hypothalamic-pituitary disconnection or after treatment with a GnRH agonist. Domest Anim Endocrinol.

[bib1.bib24] Husein MQ, Kridli RT (2002). Reproductive responses of Awassi ewes treated with either naturally occurring progesterone or synthetic progestagen. Asian Austral J Anim Sci.

[bib1.bib25] Jabbour HN, Evans G (1991). Fertility of superovulated ewes following intrauterine or oviducal insemination with fresh or frozen-thawed semen. Reprod Fertil Dev.

[bib1.bib26] Kumar S, Mishra AK, Kolte AP, Dash SK, Karim SA (2008). Screening for Booroola (FecB) and Galway (FecXG) mutations in Indian sheep. Small Rumin Res.

[bib1.bib27] Liu SF, Jiang YL, Du LX (2003). Studies of BMPR-IB and BMP15 as candidate genes for fecundity in little tailed Han sheep. Acta Genet Sin.

[bib1.bib28] Luther JS, Grazul-Bilska AT, Kirsch JD, Weigl RM, Kraft KC, Navanukraw C, Pant D, Reynolds LP, Redmer DA (2007). The effect of GnRH, eCG and progestin type on estrous synchronization following laparoscopic AI in ewes. Small Rumin Res.

[bib1.bib29] Mahdavi M, Nanekarani S, Hosseini SD (2014). Mutation in BMPR-IB gene is associated with litter size in Iranian Kalehkoohi sheep. Anim Reprod Sci.

[bib1.bib30] Maxwell WMC (1986). Artificial insemination of ewes with frozen-thawed semen at a synchronised oestrus. 1. Effect of time of Onset of oestrus, ovulation and insemination on fertility. Anim Reprod Sci.

[bib1.bib31] Maxwell WMC, Barnes DR (1986). Induction of oestrus in ewes using a controlled internal drug release device and eCG. J Agric Sci.

[bib1.bib32] Maxwell WMC, Butler LG, Wilson HR (1984). Intra-uterine insemination of ewes with frozen semen. J Agri Sci.

[bib1.bib33] Maxwell WMC, Evans G, Rhodes SL, Hillard MA, Bindon BM (1993). Fertility of superovulated ewes after intrauterine or oviducal insemination with low numbers of fresh or frozen-thawed spermatozoa. Reprod Fertil Dev.

[bib1.bib34] Menchaca A, Miller V, Salveraglio V, Rubianes E (2007). Endocrine, luteal and follicular responses after the use of the short-term protocol to synchronize ovulation in goats. Anim Reprod Sci.

[bib1.bib35] Meyer HH, Baker RL, Harvey SM, Hickey SM (1994). Effects of Booroola Merino breeding and the FecB gene on performance of crosses with longwool breeds. 2. Effects on reproductive performance and weight of lamb weaned by young ewes. Livest Prod Sci.

[bib1.bib36] Mishra AK, Arora AL, Kumar S, Singh VK (2007). Improving productivity of Malpura breed by crossbreeding with prolific Garole sheep in India. Small Rumin Res.

[bib1.bib37] Mortazavi SH, Eslami M, Farrokhi-Ardabili F (2020). Comparison of different carrier-compounds and varying concentrations of oleic acid on freezing tolerance of ram spermatozoa in tris-citric acid-egg yolk plasma semen diluent. Anim Reprod Sci.

[bib1.bib38] Mulsant P, Lecerf F, Fabre S, Schibler L, Monget P, Lanneluc I, Pisselet C, Riquet J, Monniaux D, Callebaut I, Cribiu E, Thimonier J, Teyssier J, Bodin L, Cognié Y, Chitour N, Elsen JM (2001). Mutation in bone morphogenetic protein receptor-IB is associated with increased ovulation rate in Booroola Mérino ewes. P Natl Acad Sci USA.

[bib1.bib39] Qasemi-Panahi B, Rafat SA, Ebrahimi M, Akbarzadeh MH, Hajializadeh-Valilou R (2016). New technique for activating reproductive system during non-breeding season in Ghezel ewes. Iran J Appl Anim Sci.

[bib1.bib40] Qi MY, Xu LQ, Zhang JN, Li MO, Lu MH, Yao YC (2020). Effect of the Booroola fecundity (FecB) gene on the reproductive performance of ewes under assisted reproduction. Theriogenology.

[bib1.bib41] Reader KL, Haydon LJ, Littlejohn RP, Juengel JL, McNatty KP (2012). Booroola BMPR1B mutation alters early follicular development and oocyte ultrastructure in sheep. Reprod Fertil Dev.

[bib1.bib42] Rensis DF, López-Gatius F (2014). Use of equine chorionic gonadotropin to control reproduction of the dairy cow: a review. Reprod Domest Anim.

[bib1.bib43] Reyna J, Thomson PC, Evans G, Maxwell WMC (2007). Synchrony of ovulation and follicular dynamics in merino ewes treated with GnRH in the breeding and non-breeding seasons. Reprod Domest Anim.

[bib1.bib44] Robinson TJ (1965). Use of progestagen-impregnated sponges interserted intravaginally or subcutaneously for the control of the oestrous cycle in the sheep. Nature.

[bib1.bib45] Romano JE (1996). Comparison of fluoregestone and medroxyprogesterone intravaginal pessaries for estrus synchronization in dairy goats. Small Rumin Res.

[bib1.bib46] Ryan N, Rosner A (2001). Quality of life and costs associated with micronized progesterone and medroxyprogesterone acetate in hormone replacement therapy for nonhysterectomized, postmenopausal women. Clin Ther.

[bib1.bib47] Salamon S, Maxwell WMC (2000). Storage of ram semen. Anim Reprod Sci.

[bib1.bib48] Salehi R, Kohrama H, Towhidi A, Kermani Moakhar H, Honarvar M (2010). Follicular development and ovulation rate following different superovulatory treatments in Chall ewes. Small Rumin Res.

[bib1.bib49] Souza CJ, Moraes JCF, Chagas LM (1994). Effect of the Booroola gene on time of ovulation and ovulatory dynamics. Anim Reprod Sci.

[bib1.bib50] Souza CJ, Campbell BK, Webb R, Baird DT (1997). Secretion of inhibin A and follicular dynamics throughout the estrous cycle in the sheep with and without the Booroola gene (FecB). Endocrinology.

[bib1.bib51] Souza CJ, MacDougall C, Campbell BK, McNeilly AS, Baird DT (2001). The Booroola (FecB) phenotype is associated with a mutation in the bone morphogenetic receptor type 1 B (BMPR1B) gene. J Endocrinol.

[bib1.bib52] Viñoles C, Meikle A, Forsberg M, Rubianes E (1999). The effect of subluteal levels of exogenous progesterone on follicular dynamics and endocrine patterns during early luteal phase of the ewe. Theriogenology.

[bib1.bib53] Walker SK, Smith DH, Godfrey B, Seamark RF (1989). Time of ovulation in the South Australian Merino ewe following synchronization of estrus. 1. Variation within and between flocks. Theriogenology.

[bib1.bib54] Wilson T, Wu XY, Juengel JL, Ross IK, Lumsden JM, Lord EA, Dodds KG, Walling GA, McEwan JC, O'Connell AR, McNatty KP, Montgomery GW (2001). Highly prolific Booroola sheep have a mutation in the intracellular kinase domain of bone morphogenetic protein IB receptor (ALK-6) that is expressed in both oocytes and granulosa cells. Biol Reprod.

[bib1.bib55] Yao YC, Tian L, Han HB, Chen XH, Lu MH, Guo PC, Zhang CF, Li N, Lian ZX, Li W (2006). Preponderance genotype of BMPR-IB improves the pregnant rate of embryo-transfer in sheep. Prog Biochem Biophys.

